# Microstructure and Mechanical Properties of 4Al Alumina-Forming Austenitic Steel after Cold-Rolling Deformation and Annealing

**DOI:** 10.3390/ma13122767

**Published:** 2020-06-18

**Authors:** Chenchen Jiang, Qiuzhi Gao, Hailian Zhang, Ziyun Liu, Huijun Li

**Affiliations:** 1 School of Resources and Materials, Northeastern University at Qinhuangdao, Qinhuangdao 066004, China; jiangchen_ly@163.com (C.J.); hhhliuziyun@163.com (Z.L.); 2 School of Materials Science and Engineering, Northeastern University, Shenyang 110819, China; 3 Daotian High Technology Co., Ltd., Qinhuangdao 066004, China; 4 School of Materials Science & Engineering, Tianjin University, Tianjin 300354, China; huijun@uow.edu.au

**Keywords:** alumina-forming austenitic steel, cold rolling, dislocation density, mechanical properties

## Abstract

Microstructural evolutions of the 4Al alumina-forming austenitic steel after cold rolling with different reductions from 5% to 30% and then annealing were investigated using electron backscattering diffraction (EBSD), X-ray diffraction (XRD) and transmission electron microscopy (TEM). Tensile properties and hardness were also measured. The results show that the average grain size gradually decreases with an increase in the cold-rolling reduction. The low angle grain boundaries (LAGBs) are dominant in the cold-rolled samples, but high angle grain boundaries (HAGBs) form in the annealed samples, indicating that the grains are refined under the action of dislocations. During cold rolling, high-density dislocations are initially introduced in the samples, which contributes to a large number of dislocations remaining after annealing. With the sustaining increase in cold-rolled deformation, the samples exhibit more excellent tensile strength and hardness due to the decrease in grain size and increase in dislocation density, especially for the samples subjected to 30% cold-rolling reduction. The contribution of dislocations on yield strength is more than 60%.

## 1. Introduction

As a high-temperature alloy, since it was firstly developed at Oak Ridge National Laboratory (ORNL) in 2007, alumina-forming austenitic heat-resistant (AFA) steel hopefully, instead of the ferritic steel and nickel-based alloy, has enormous potential to be applied to ultra-supercritical thermal power plants [1,2,3]. AFA steel is very promising for application in aggressive high-temperature steam in excess of 700 °C to promote energy conversion efficiency owing to the considerable creep life and excellent oxidation resistance of AFA steel. The additions of Ni, Al, Nb and Mo elements have facilitated the formation of many kinds of precipitates in the austenitic matrix of AFA steel, such as MC-type carbides, Laves, B2-NiAl and γ′-Ni_3_Al phase particles [4,5]. Generally, there are mainly two approaches that have been reported to strengthen AFA steel depending on these precipitates [1]: First, pinning the dislocations through the diffusely distributed second phases with small sizes to improve the creep strength. Second, enhancing the oxidation resistance by means of homogeneous and dense Al_2_O_3_ scale whose continuous formation on the surface of AFA steel may be supported by the B2-NiAl phase [6].

Yamamoto et al. [7] found that nano-sized Laves-Fe_2_Nb and Ni_3_Al dispersions, particularly around 100 nm in diameter, were effective at improving the creep resistance. Zhao et al. [8] researched the coarsen kinetics of precipitates during aging and discovered the secondary NbC with thermal stability, which enhanced creep life of AFA steel by about 4500 h at 750 °C/100 MPa. The desired results for AFA steel are not only attributed to the designed composition but also reasonable pre-treating methods. It has been reported that cold work with high deformation before aging can accelerate the precipitation and visibly augment the quantity of precipitated phases to increase the deformation resistance [9,10]. Additionally, the nucleation positions of dislocations were introduced to significantly prolong the creep life by imposing low pre-strain below 10% on AFA steel.

However, it should not be neglected that, as the component in the matrix of austenitic stainless steel, austenitic grain boundaries play an indispensable role regardless of acting as dislocation sources or barriers to hinder dislocation migration [11,12,13]. Using the approach of cold-rolled deformation, the different distributions of grain boundaries and dislocations can be achieved to control the properties of metals. The Hall–Petch formula has been proved that great refinement of grains even to nanoscale can remarkably improve strength of materials [14]. Xu et al. [15] obtained small and uniform grains through cold rolling the 18Cr-8Ni stainless steel by 90% and then annealing. Hughes et al. [16] affirmed that the high-angle grain boundaries along with low-angle grain boundaries can contribute to enhance the strength properties of Ni metal through large deformation. Wang et al. [17] applied different stress-strain conditions to 304L stainless steel and observed a variation of dislocation density. Furthermore, Dini et al. [18] claimed that the twin boundaries were obstacles to pin the migration of dislocations, resulting in the instantaneous enlargement of the work hardening rate. These previous works have researched the characterization of grain boundaries and dislocations, which is equally important but less for AFA steel.

In the present paper, the effect of cold rolling on AFA steel has been researched after reducing the samples by different thicknesses and then annealing at 1150 °C for 30 min. The prime interest is to survey the microstructural evolutions including grain boundaries and dislocations, which were investigated by electron backscattering diffraction (EBSD), X-ray diffraction (XRD) and transmission electron microscopy (TEM). Besides, the mechanism of the changes in mechanical properties of the AFA steel was discussed in detail. The investigation of microstructure and mechanical properties after cold rolling will help us to conduct appropriate creep tests for advancing high-temperature application of AFA steel.

## 2. Experimental Procedures

The original material constituted of 99.9% pure metals was arc melted in a vacuum atmosphere and drop cast to an ingot to prepare for AFA steel. The chemical composition (mass%) of AFA steel is listed in Table 1, which was measured by inductively coupled plasma-optical emission spectrometry (ICP-OES, Agilent, Palo Alto, CA, USA). In order to improve the structural defects, such as solute segregation and the central pipe, the ingot was homogenized at 1180 °C for 5 h and hot rolled to a 10 mm thick sheet at 1250 °C after homogenization, then cooled at room temperature. Subsequently, the sheet was cut into six cuboids with a size of 10 mm × 8 mm × 80 mm by a wire-cutting machine along the hot rolling direction. The cuboids were deformed with thickness reduced by 5%, 10% and 30% at room temperature, respectively. After that, half of cold-rolled cuboids were isothermally annealed at 1150 °C for 30 min and cooled in a muffle furnace.

The samples subjected to cold rolling and annealing were cut to 5 mm × 5 mm × 6 mm for investigation in microstructure via EBSD study. A series of SiC papers from 320 grit to 2000 grit were used for mildly polishing all samples, and before light etching by OP-S silica solution, a pre-polish using diamond pastes was applied. A Schottky field emission gun scanning microscope (JEOL-JSM7001F, Tokyo, Japan) equipped with a Nordlys-II (S) detector was applied to EBSD analysis, whose acceleration voltage and current in probe were 30 kV and 10 mA, respectively. A working distance of 24 mm was set. The obtained data from the EBSD maps were processed by the Oxford Instruments Channel-5^TM^ Software (Oxford Instruments, Oxford, UK). The phase structure and dislocation density analysis were explored by XRD using Cu Kα radiation (Rigaku, Tokyo, Japan) with scanning speed of 4°/min and scanning range from 20° to 100°. TEM analysis was performed on a field-emission electron microscope FEI Tecnai G2F20 (Hillsboro, OR, USA) working at 200 kV and thin foils with 3 mm in diameter and 80–100 μm in thickness were prepared and then treated by ion milling with argon ions.

**Figure 1:**
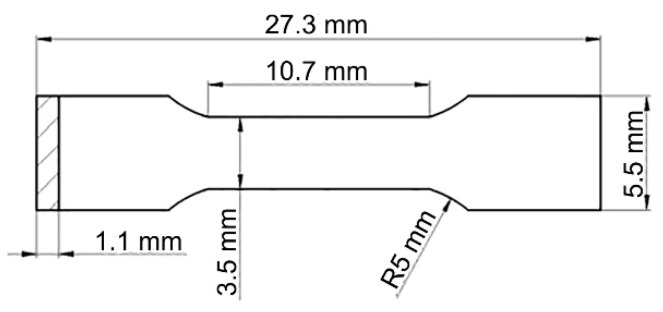
Shape of the tensile samples.

For the purpose of exploring the effects of microstructure, the mechanical properties of AFA steel were examined. In order to decrease the error of the experiment, each group of three tensile samples was prepared for the AFA steel with different cold-rolled reduction. Tensile testing was conducted at a tensile rate of 0.3 mm/min at room temperature, using the designed samples in Figure 1. Vicker’s hardness testing was carried out under a load of 49 N for 10 s, and the final values of hardness were acquired by averaging 10 measurements of each group.

## 3. Results

### 3.1. Microstructural Evolution

**Figure 2:**
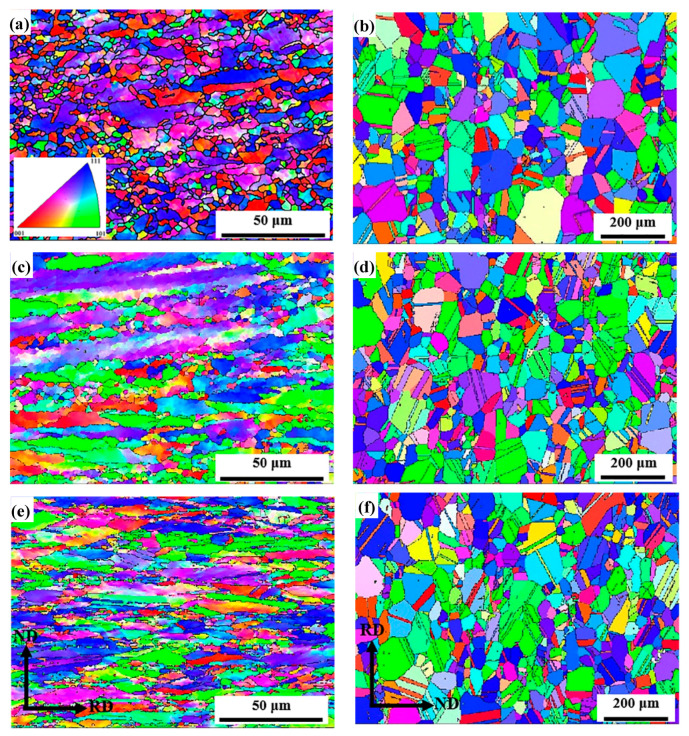
Orientation imaging maps of the AFA steel. (**a**), (**c**), (**e**) represent cold-rolled samples with reductions of 5%, 10%, 30%, and (**b**), (**d**), (**f**) represent the corresponding annealed samples after cold rolling, respectively.

Figure 2 shows orientation imaging maps of the AFA steel after cold rolling and annealing, respectively. The grain boundaries and orientation are displayed by different color patterns, and the same color represents the accordant orientation of the crystalline grains. The grains of cold-rolled samples typically elongate along the rolling direction (RD) because of the increase in deformation (Figure 2a,c,e) and still have different orientation features principally expressed by three colors. By increasing the cold-rolling reduction to 30%, the grain size decreases significantly. Moreover, the grain orientation gradually turns to the direction of <101> (green color) in Figure 2c. During annealing at 1150 °C for 30 min, the grains were increasingly equiaxed, and recovery and recrystallization occurred in the cold-rolled samples (Figure 2b,d,f). In order to analyze microstructural evolutions of the experimental steel, quantitative data were collected by counting number of different grains from the orientation imaging maps. The obtained results are listed in Table 2. The fraction of deformed grains keeps improving with the increase in cold-rolling reduction. In addition, almost all the cold-rolled samples have recrystallized after annealing. It should be noted from Figure 2b,d,f that a large number of twin boundaries can be seen in the annealed samples. These annealed twin boundaries can play an important role in hindering dislocation migration to improve the mechanical properties of materials.

**Figure 3:**
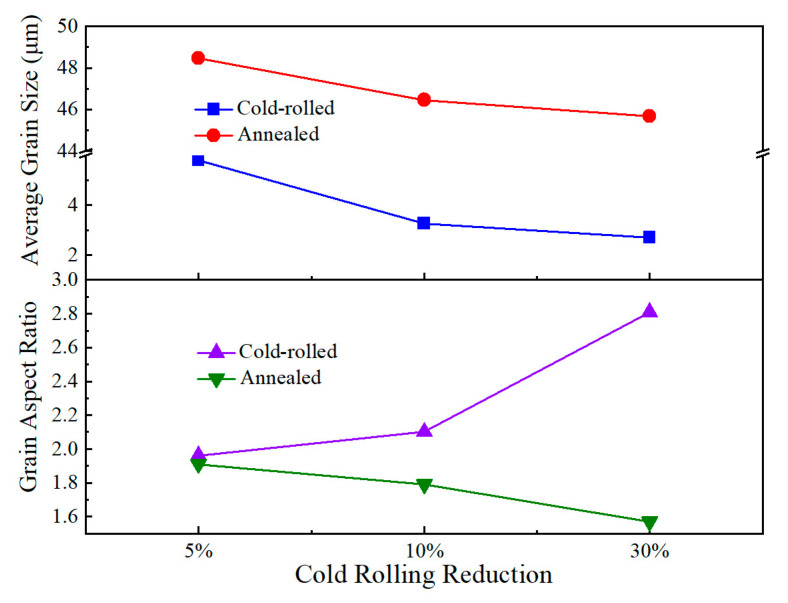
Average grain size and grain aspect ratio of the samples as a function of cold-rolling reduction.

The average grain size and grain aspect ratio of the samples based on quantitative description from EBSD data as a function of cold-rolling reduction are depicted in Figure 3. It is clear that the average values of grain size are all less than 6 μm, which decreases from 5.79 μm to 2.7 μm with increasing cold-rolling reduction from 5% to 30% prior to annealing at 1150 °C for 30 min. Obviously, the grain aspect ratio has an evident growth trend due to the further cold-rolled deformation. The grain size of annealed samples smoothly goes down from 48.46 μm to 45.67 μm. The sample with 30% reduction also possesses the lowest grain aspect ratio of 1.57.

**Figure 4:**
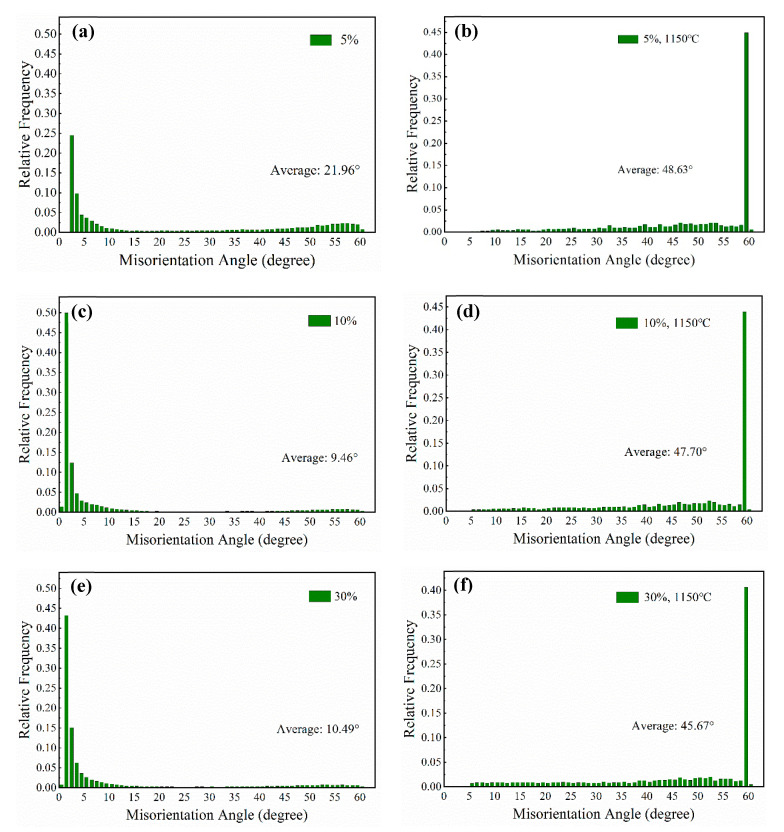
Misorientation angle distributions of the samples reduced by (**a**) 5%, (**c**) 10% and (**e**) 30% and then annealed at 1150 °C for 30 min after cold rolling (**b, d, f**), respectively.

Figure 4 shows misorientation angle distributions of the AFA steel after cold rolling and annealing, respectively. LAGBs with misorientation angle below 15° are dominant after cold rolling at room temperature compared to that in the as-received sample investigated in our previous work [19]. Firstly, the statistical value of LAGBs is 53.8% in proportion and then takes a dramatic growth to 84%, following by decreasing to 81% with the increase in cold-rolled reduction from 5% to 30%. However, the annealing treatment at 1150 °C for 30 min greatly affected the reaction and migration of grain boundaries [20]. The LAGBs mostly transformed into HAGBs which presented a peak at 59° in Figure 4b,d. Furthermore, the average misorientation angle of 45.67° was lowest and more LAGBs existed in the sample reduced by 30% and then annealed (Figure 4f).

### 3.2. Dislocation Density and Characterization

**Figure 5:**
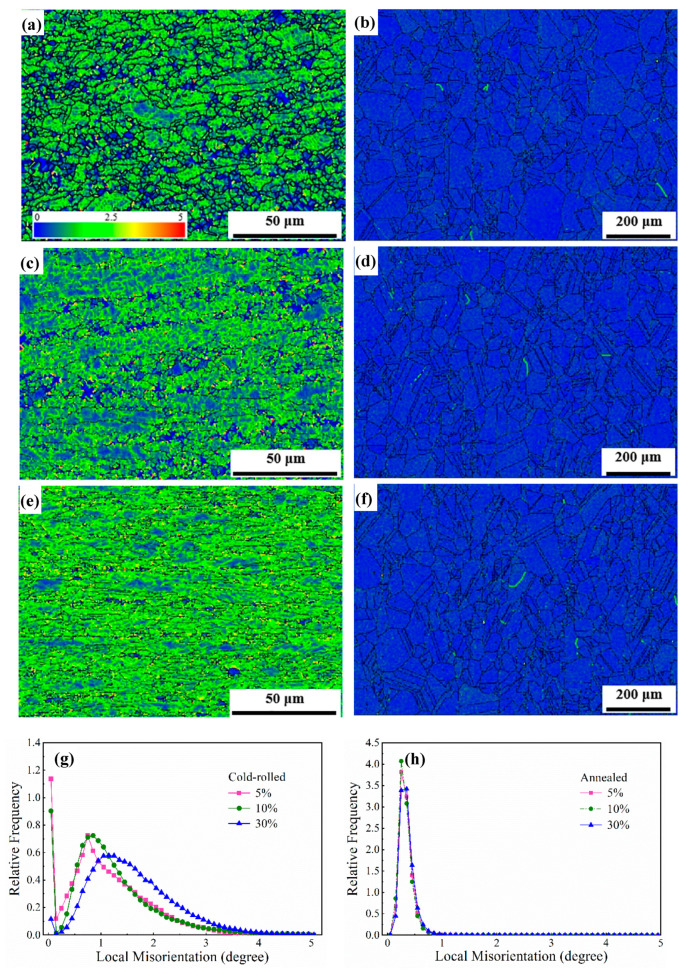
Local misorientation distributions of the AFA steel reduced by (**a**) 5%, (**c**) 10% and (**e**) 30% and then annealed at 1150 °C for 30 min after cold rolling (**b**, **d**, **f**). (**g**) and (**h**) represent curves of local misorientation distributions, respectively.

The dislocation density can be measured by local misorientation obtained via scanning with step sizes of 0.3 μm in cold-rolled samples and 2 μm in annealed samples, respectively [21,22]. The local misorientation distribution images and curves obtained are presented in Figure 5. It should be pointed that 5° was set as the threshold value to avoid the error caused by LAGBs. In fact, the local misorientation mainly distributes below 3.5° after cold rolling from Figure 5g. Distinctly, the local misorientation in grains presents inhomogeneous distributions and gradually increases, particularly around the grain boundaries (covered by yellow color) (Figure 5a,c,e). The larger misorientation around grain boundaries may result from the formation of geometrically necessary dislocations during plastic deformation [23,24]. In Figure 5g, it can be also found that local misorientation angle distributions totally increase with increasing the cold deformation reduction, and the peak values appear at 0.75°, 0.85° and 1.25°, respectively, which means the average value of misorientation angle increases. After annealing at 1150 °C for 30 min, almost all of the local misorientation is below 1° (Figure 5h), which is attributed to the disappearance of dislocations at high temperature.

**Figure 6:**
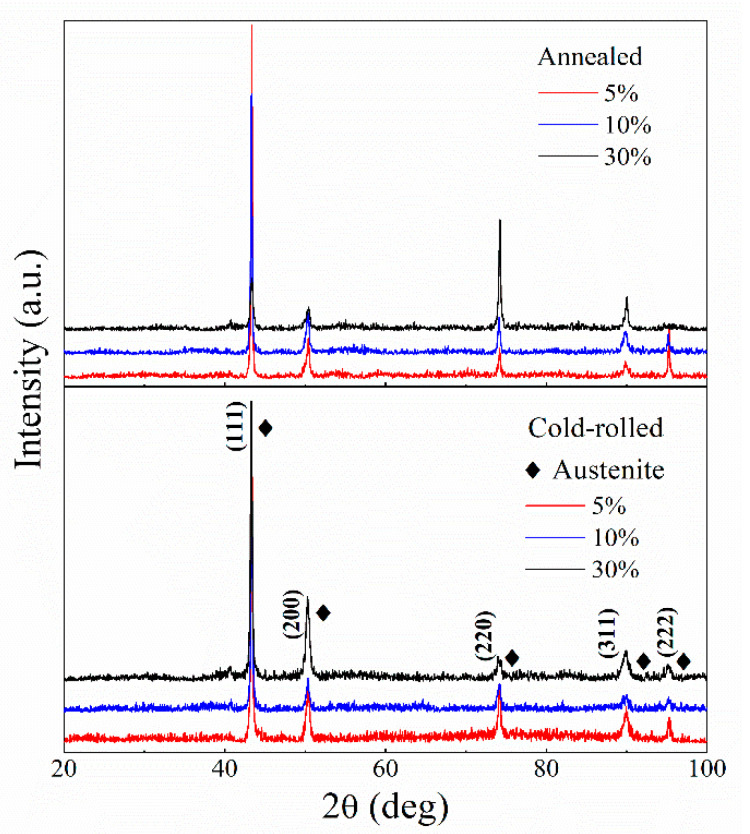
X-ray diffraction pattern of the AFA steel subjected to cold rolling at room temperature and annealing at 1150 °C for 30 min, respectively.

In order to quantitatively evaluate the dislocation density, XRD patterns of the samples are shown in Figure 6. It can be seen that the clear diffraction peaks recognized as austenite are the crystal planes of (111), (200), (220) and (311), respectively, which can be considered as the primary source of dislocation. Based on the Williamson–Hall method, the grain size and microstrain can cause XRD peaks broadening. In practice, the full width at half maximum (FWHM) is used to represent the peak width, which is related to the strain of material. FWHM can be solved by using the integral method through making a tangent line at the bottom of the peak to measure the area and height, and then dividing both of them. The relevant information listed in Table 3 was obtained by MDI Jade software analysis. Then, dislocation density (*ρ*) can be calculated by the following equation [25]:

(1) $$\rho =14.4\frac{\varepsilon^{2}}{b^{2}}$$

where *ε* is the microstrain, *b* is the burgers vector ($b=a/\sqrt{2}$, a = 0.36 nm for AFA steel with face-centered cubic structure). The dislocation density calculated from XRD provides a numerical reference although it is not quite an accurate value. The cold rolled treatment causes the change in dislocation density. For example, the value of dislocation density in 30% cold-rolled sample increases to 8.02 × 10^14^ m^−2^ from 3.2 × 10^14^ m^−2^ in 5% cold-rolled sample. Because of the increase in prior cold-rolled reductions, the samples also preserve more dislocations with the annihilation of partial dislocations after annealing. The dislocation density is on the same order of magnitude than that in 10% and 30% cold-rolled samples.

**Figure 7:**
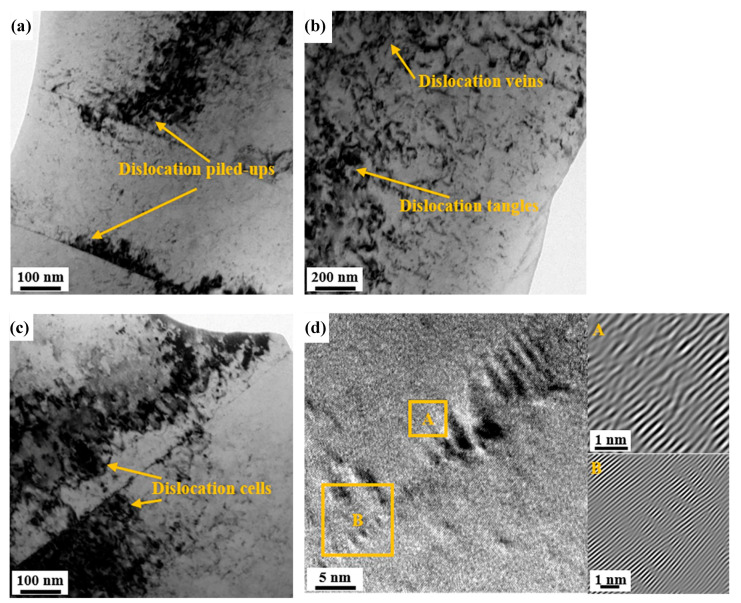
Transmission electron microscope (TEM) images of the AFA steel with 30% cold rolling reduction. (**a**) Dislocation piled-ups, (**b**) dislocation veins and tangles, (**c**) dislocation cells and (**d**) high-resolution TEM (HRTEM) of (**c**) revealing the dislocation characters nearby the grain boundary by inverse fast Fourier transform (IFFT) of Areas A and B.

**Figure 8:**
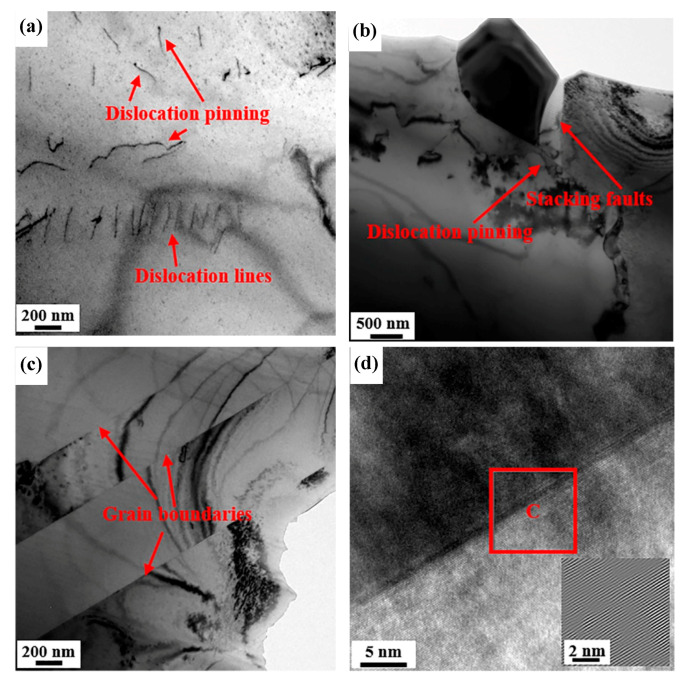
TEM images of the AFA steel annealed at 1150 °C for 30 min after 30% cold deformation. (**a**) Dislocation lines and pinning, (**b**) dislocation piled-ups and stacking faults, (**c**) annealing twin boundaries and (**d**) HRTEM of (**c**) with IFFT of Area C.

The dislocation features of the cold-rolled sample and annealed sample with 30% reduction, conducted by TEM observation, are presented in Figure 7 and Figure 8. Compared with the macro and average data obtained by XRD, TEM images illustrate more detailed and partial changes in dislocation density and structures, which can explain the differences in microstructure. Some studies proved that the results from TEM can be supported by XRD below medium or low deformation [26,27]. During cold rolling, it produced complicated dislocation structures. The dislocation piled up and even formed cellular substructures near the deformed grain boundaries (Figure 7a,c). In addition, it can be observed that some dislocation veins and tangles interweave to interact in Figure 7b. The features are caused by the inconsonant plastic deformation among grains with different orientations. Figure 7d shows the high-resolution image (HRTEM) of dislocation near the grain boundary accompanying inverse fast Fourier transform (IFFT) of Area A and B subjected to a filtering process, revealing the atoms arrange in a disordered manner. After annealing, the structures of dislocations in 30% cold-rolled sample evolve into relatively simple planar dislocation structures, which are characterized by plane dislocation lines, pinned dislocations by precipitated particles and stacking faults (Figure 8a,b). The IFFT picture of grain boundaries after annealing indicates less dislocations than that in Figure 7d.

### 3.3. Schmid Factor and Mechanical Properties

Plastic deformation can occur in metals and alloys under external force. The Schmid factor (SF) can be used to estimate the capacity of deformation resistance, that is yield strength [28]. Equation (2) can reflect the relationship [29]:

(2) $$\sigma_{s}=\frac{\tau_{c}}{m}$$

where *σ_s_* is yield stress, *τ_c_* is critical resolved shear stress, and *m* represents Schmid factor. Generally, the samples with larger SF firstly yield if the slip system operates under the same external conditions [30].

**Figure 9:**
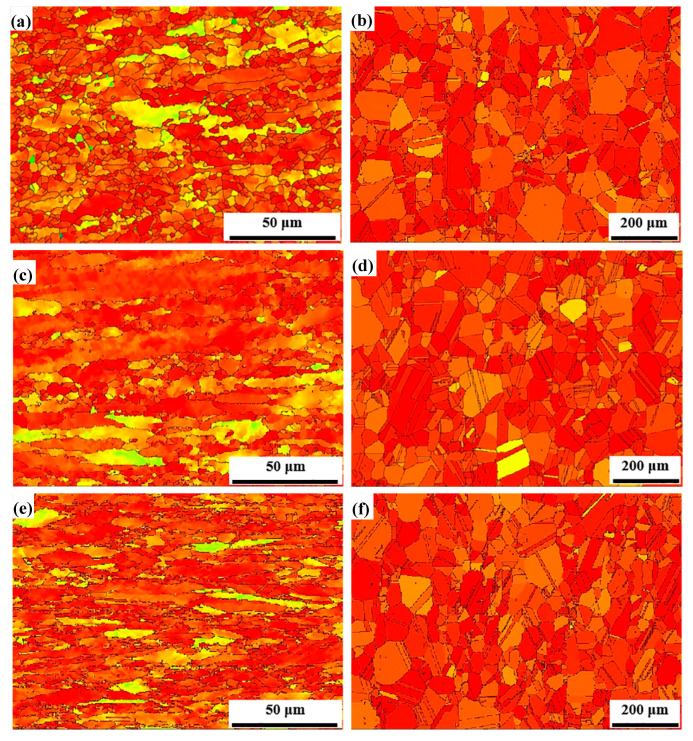
Schmid factor distributions of the AFA steel. (**a**), (**c**), (**e**) represent cold-rolled samples with reductions of 5%, 10%, 30%, and (**b**), (**d**), (**f**) represent the corresponding annealed samples after cold rolling.

**Figure 10:**
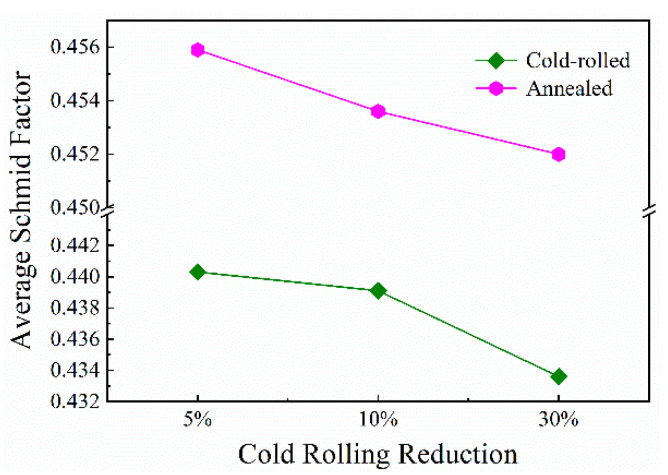
Average Schmid factor as a function of cold-rolling reduction for cold-rolled and annealed samples.

Figure 9 exhibits the grain Schmid factor distributions of the samples with different treatment conditions. The color of deformed grains is close to yellow, while the smaller equiaxed recrystallized grains are almost covered by red color, indicating the size of SF. However, in the samples annealed at 1150 °C for 30 min, in Figure 9b,d,f, the cold rolling leads some grains with yellow color to remain in hard orientation (the SF is generally below 0.25), the angle between which and the external stress is away from 45°. The slip deformation is difficult in such grains. Moreover, the average SF with variation is described by two broken lines in Figure 10. The average SF can take into account the whole load state of samples during mechanical testing. It can be seen that the average values of all samples are more than 0.432, and even beyond 0.45 in annealed samples because of the low strain and annealing at high temperature. Taken as a whole, with the increase in deformed reduction, the average SF decreases. The smaller value of SF means greater stress is necessary to make crystals slip.

**Figure 11:**
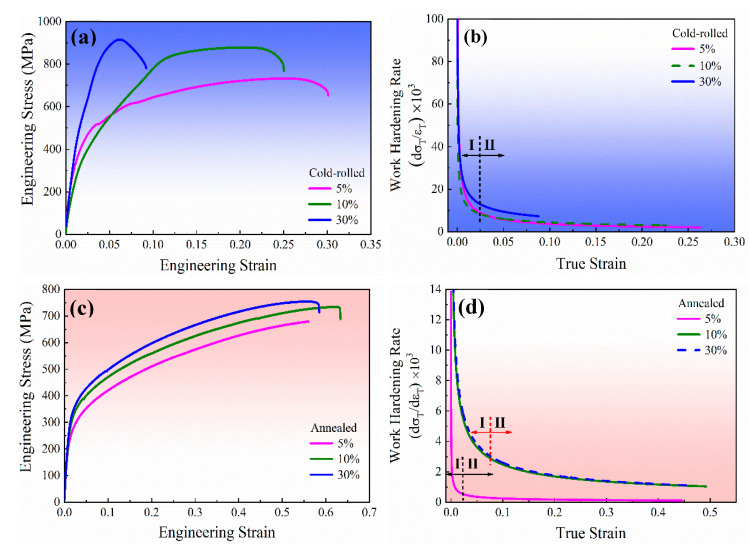
Engineering stress-strain curves (**a** and **c**), and the corresponding work hardening rate curves (**b** and **d**) with true strain of the AFA steel after cold-rolled deformation and annealing. σ_T_ and ε_T_ represent true stress and true strain, respectively.

The engineering stress-strain curves are presented in Figure 11a,c. The samples with 30% reduction intuitively display higher tensile strength both in cold-rolled and annealed samples. For the cold-rolled samples in particular, the tensile strength increases from 803 MPa to 916.2 MPa with increasing the reduction to 30%. Meanwhile the yield strength increases from 636 MPa to 909 MPa. The corresponding work hardening rate (WHR) curves based on tensile data are depicted in Figure 11b,d. The values of WHR are determined by Hollomon’s equation. It can be distinguished that the WHR curves are obviously divided into two stages as marking with Ⅰ and Ⅱ. For stage Ⅰ, the WHR decreases monotonically and quickly, and thus this stage is named as yield stage. The 5% cold-rolled sample shows higher yield resistance than the 10% cold-rolled sample in stage Ⅰ (Figure 11b), which may attribute to that the more fraction of substructures in the sample with 5% reduction can increase resistance of the micro deformation. For stage Ⅱ (macro plastic stage), a stable trend of work hardening can be observed with the variety of true strain. It should also be noted the samples with 5% cold-rolled reduction present the fastest plastic deformation with the increase in true strain during stage Ⅱ. Furthermore, compared to the samples only rolled at room temperature, the annealed samples have an advantage on elongation with a promotion no less than 30% (Table 4). The softened orientation and lowest dislocation density are the main reasons.

**Figure 12:**
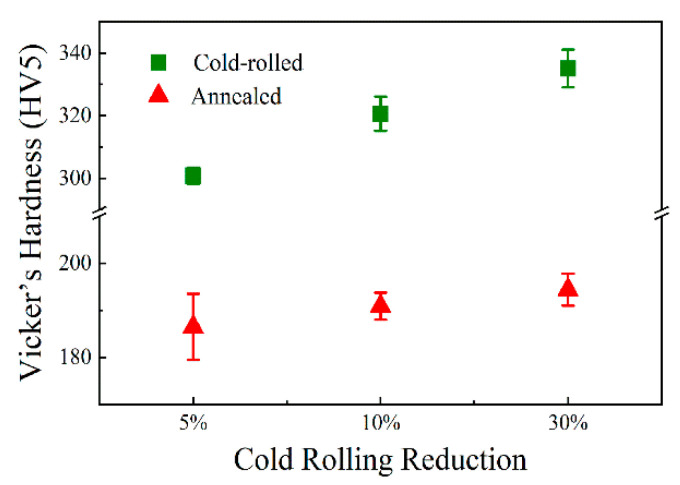
Vicker’s hardness of the AFA steel as a function of cold-rolling reduction for cold-rolled and annealed samples.

The variation of Vicker’s hardness of the samples subjected to cold-rolled deformation and annealing treatment is presented in Figure 12. The standard deviations of hardness values are 2.6, 5.4 and 6 for 5%, 10% and 30% cold-rolled samples, and 7, 2.9 and 3.4 for annealed samples, respectively. The average values of cold-rolled samples are 300.79 HV, 320.64 HV and 335 HV, respectively. In comparison to the sample with 5% cold deformation (186 HV), the samples with reductions of 10% and 30% subsequently add to the hardness by 5 HV and 9 HV, respectively, after annealing at 1150 °C for 30 min. Notably, the variation of hardness is evident to be a positive correlation with the increase in cold-rolling reduction (Figure 12). Graça et al. [31] gave the relationship between the dislocation density and hardness, and suggested that dislocations provided the material with the most resistance during the indentation taking the shape. The dislocations entangled in cold-rolled samples and were impeded by a large number of grain boundaries, resulting in difficult movement for dislocations during deformation. In this way, the increase in dislocation density can be considered as the reason for the improvement of Vicker’s hardness. Therefore, the Vicker’s hardness of cold-rolled samples is integrally higher than that of annealed samples.

## 4. Discussion

### 4.1. The Effect of Cold Rolling on Microstructural Evolution

Through the results investigated from above, it is quite important for the AFA steel to assess the effect of cold rolling on the microstructural evolutions during the deformation and annealing. In this study, the AFA steel is an austenitic stainless steel in essence. The structure features caused by cold rolling depend on the stacking fault energy (SFE) of austenitic steels to a great extent [32,33]. The SFE of traditional austenitic steels is usually less than 100 mJ/m^2^. Schramm et al. [34] have given the estimation method of SFE in some commercial austenitic steels, which can be defined by the following relationship:

(3) $$\gamma =-53+6.2\omega_{Ni}+0.7\omega_{Cr}+3.2\omega_{Mn}+9.3\omega_{Mo}$$

where *γ* (mJ/m^2^) is the value of SFE, *ω_X_* represents the height percent of elements. Because the content of aluminum element is not less than that of molybdenum, the SFE of AFA steel is reckoned to be more than 109 mJ/m^2^. This can account for deformed mechanism during cold rolling and the formation of twin boundaries after annealing [35].

With the increase in strain, the matrix was divided into lots of new structural blocks with the grain boundaries splitting. It can be seen that a large proportion of LAGBs are approximately at 2.5° in Figure 4a,c,e, illustrating the formation of sub-grains. However, when the sample was reduced by 30% at room temperature, the average misorientation angle has a small increase to 10.49° (Figure 4e), which indicates that the content of HAGBs increases. The local misorientation indirectly reflects the change in dislocation density which is also proved by XRD (Figure 5 and Table 3). In the process of increasing strain, it gradually produced high-density dislocation entanglements and even cellular structures with a geometric interface. The high stress concentration around grain boundaries could prompt sub-boundaries to turn to grain boundaries with higher angles. The boundaries were formed by the cross-slip of dislocations [36,37,38]. During cold-rolled deformation, some dislocations integrated into boundaries, and some dislocations converged and entangled around boundaries. Based on the theory of strain gradient plasticity, some researchers suggested that the boundaries under sustaining stress could transform into geometrically necessary boundaries (GNBs) and incidental dislocation boundaries (IDBs), which could be also regarded as the extended dislocation boundaries [16,36]. GNBs were straight with a certain macro orientation and a large deviation between the interface of both sides [39]. During the process of formation, the grains were subdivided. IDBs with a small misorientation are generally formed by capturing the dislocations during the interaction between pinned dislocations and slip dislocations activated by plastic deformation [40]. At low and medium (5% and 10%) deformation, GNBs and IDBs were more likely to be LAGBs. Nevertheless, for a larger deformation of 30%, some GNBs shifted to the rolling direction with the rotation of grains, resulting in the increase in the fraction of HAGBs.

On the other hand, after cold rolling, plenty of dislocations that were stored in cells contributed to form the nuclei of recrystallization. The increasing positions of nucleation promoted the refinement of grains. Because of the migration of HAGBs, lots of annealed twin boundaries formed during annealing treatment. The phenomenon can explain the appearance of peak values at around 59° in Figure 4b,d,f. In the coincidence site lattice (CSL) model, the misorientation angle was proved to be the special high angle grain boundaries of ∑3 [41]. The orientation relationship of ∑3 revealed that the misorientation between annealing twins and maternal grains was <111>/60°. Because some dislocations decomposed and glided into HAGBs through the annealing treatment, part of the dislocations were consumed. Owing to the prior cold-rolled deformation, lots of dislocations that were undecomposed and remained after decomposing arranged in loose structures in annealed samples (Figure 8).

### 4.2. Grain Boundary Strengthening and Dislocation Strengthening

The results reported in this paper are in pursuit of determining the effects of cold rolling on microstructure and mechanical properties. The properties intensively depend on the changes in microstructure of materials [15,42]. Grain boundaries migration and dislocations slipping were the primary mechanism of plastic deformation during mechanical testing in accordance with the SFE of AFA steel. The mechanism of grain boundary strengthening can be explained using the Hall–Petch relationship. As the cold rolling reduction increased, the grain aspect ratio also increased with the decrease in boundaries’ spacing in the cold-rolled samples. In addition, the grains grew up and approached a condition of being equiaxed after annealing. The classical Hall–Petch can conduct the strengthening from grain boundaries when the coordinated deformation of grains occurs at external force, which is given as [43,44]:

(4) $$\sigma_{s}=\sigma_{0}+kD_{GB}^{1/2}$$

where *σ_0_* and *k* are constants. σ_0_ shows the deformation resistance from the interior of grains. *D_GB_* is the average grain size. This equation expresses that the grain size has an effect on yield point of the alloy. The Hall–Petch coefficient, *k*, represents the effect of grain boundaries on strength and the value is 395 MPa μm^1/2^ [45]. Therefore, the contribution of grain boundary strengthening (*σ_GB_*) can be assessed (Table 5). For cold-rolled samples with 5%, 10% and 30% reductions, the strength values from grain boundaries are 164.16 MPa, 218.77 MPa and 240.39 MPa, respectively. For the different annealed samples’ reductions, the values are 56.74 MPa, 57.95 MPa and 58.45MPa, respectively. Hence the cold-rolled samples showed higher strength compared with the annealed samples, and the samples with lager reduction also exhibited higher strength. Practically, the proliferative dislocations were hindered by the obstacle of grain boundaries and had difficulty migrating until the resistance of boundaries reached the limit, which was the so-called dynamic Hall–Petch strengthening during tensile deformation [46]. Notably, the special ∑3 boundaries with low energy not only made a contribution to increase the content of total boundaries, but also prevented the failure behavior through blocking the continuity of common HAGBs [47].

The dislocation density both in cold-rolled and annealed samples increased with the increase in cold-rolling reduction, and the tensile and yield strength also increased similarly. Therefore, the enhancement of strength can also be considered as the result from the contribution of dislocations. In order to make the strengthening level specific, it can be conservatively presented in the form [42]:

(5) $$\sigma_{d}=M\alpha Gb\sqrt{\rho }$$

where *M* is the Taylor factor (for the AFA alloy, *M* ≈ 2.2), *α* is a constant of material, which is usually between 0.2–0.5, *G* is the shear modulus (80 GPa) and *ρ* is the dislocation density. It can be seen that the value of *σ_d_* is related to the dislocation density in materials, which was be calculated and listed in Table 5. *σ_d_* increases from 393.55 MPa to 623.12 MPa as the reduction increases from 5% to 30%. Similarly, *σ_d_* for annealed samples increases from 203.31 MPa to 249.29 MPa. The higher-density dislocations positively contribute more on strengthening the AFA steel. In the course of tensile deformation, an increasing number of dislocations proliferated and came into being as tangles around boundaries, leading to the larger deformation resistance. Hence, the samples with 30% reduction must be under enough stress to force the cumulate dislocations to slip and even cross grain boundaries.

As can be seen from Table 5, the deformation resistance mainly comes from two aspects during the tensile testing. One is the interaction between the grain boundaries and dislocations. The other is that the dislocations impede themselves from migration. Moreover, the precipitated particles can also pin the dislocations to strengthen the AFA steel. However, because the number of precipitates is few, the effect of precipitation strengthening can be ignored in the study. Therefore, it is obvious that the promotion of strength with the increase in cold rolling reduction jointly results from grain boundary strengthening and dislocation strengthening. In particular, the contribution of dislocations on strength is more than 60%. Although the results of the two strengthening mechanisms are weakened due to recrystallization and the disappearance of some dislocations during the annealing treatment, the importance of cold-rolled deformation is still clearly presented. The AFA steel after strengthening exhibits good strength and hardness when compared to some other AFA-type alloys [48,49]. It is worth conducting more systematic research on cold rolling to improve the properties of the AFA steel.

## 5. Conclusions

The microstructure evolutions of the AFA steel subjected to different cold-rolled reductions from 5% to 30% and then annealing were investigated. The mechanical properties were also measured. The main conclusions can be described as follows:

(1) The microstructure of AFA steel was significantly affected by the cold rolling. With the increase in cold-rolling reduction, the average grain size decreased. After annealing treatment, the grain aspect ratio declined, grains were gradually equiaxed and lots of twin boundaries formed.
(2) Most grain boundaries were LAGBs which distributed below 15° after cold rolling, and the HAGBs were dominant and appeared as peaks around 59° after annealing treatment.
(3) The dislocation density increased from 3.2 × 10^14^ m^−2^ to 8.02 × 10^18^ m^−2^ by increasing the reduction from 5% to 30%. The dislocations tangled, piled up and even formed cellular substructures. After annealing treatment, some stored dislocations decomposed and annihilated, and the dislocation density integrally decreased. However, the dislocation density was highest in the sample with 30% reduction.
(4) Grain boundaries and dislocation strengthening mechanisms are dominant for the cold-deformed AFA steel. With the increase in cold-rolled deformation, the strength and hardness presented an increased trend in cold-rolled and annealed samples. The highest yield strength and hardness were 909 MPa and 335 HV of 30% cold-rolled samples, respectively. The strength properties decreased after annealing. The contribution of dislocations on strength was more than 60% in all samples.

## Figures and Tables

**Table 1:** Chemical composition of alumina-forming austenitic heat-resistant (AFA) steel (mass%).

Element	Fe	Cr	Ni	Al	Si	Mo	Nb	C	Mn	W	Cu	Ti	P
wt.%	Bal	11.16	20.54	3.96	0.14	2.25	2.02	0.06	2.06	0.05	0.05	0.013	0.04

**Table 2:** Fraction of various grain types in cold-rolled samples (5%, 10% and 30%) and annealed samples (%).

Sample	Cold-Rolled			Annealed		
	5%	10%	30%	5%	10%	30%
Recrystallized	3.2	1.13	1.75	96.80	97.21	97.56
Substructured	23.15	8.91	1.98	2.98	2.59	2.28
Deformed	73.65	89.96	96.26	0.22	0.20	0.16

**Table 3:** Microstrain and dislocation density in the samples of AFA steel.

Sample	Cold-rolled			Annealed		
	5%	10%	30%	5%	10%	30%
*ε* (%)	0.12	0.13	0.19	0.062	0.07	0.129
*ρ* (10^14^ m^−2^)	3.2	3.76	8.02	0.854	1.08	1.25

**Table 4:** Tensile properties of the AFA steel subjected to cold-rolled deformation and annealing treatment, respectively.

Sample	Cold-Rolled			Annealed		
	5%	10%	30%	5%	10%	30%
Yield strength (MPa)	636	703	909	321.6	374	396
Tensile strength (MPa)	803	879.5	916.2	680.15	734.6	755
Elongation (%)	30	25.3	10.3	63.6	58.9	56

**Table 5:** Contributions of grain boundaries and dislocation on strength. P*σ_GB_* and P*σ_d_* represent the proportion of grain boundary and dislocation strengthening in yield strength, respectively.

Sample	Cold-Rolled			Annealed		
	5%	10%	30%	5%	10%	30%
*σ_GB_* (MPa)	164.16	218.77	240.39	56.74	57.95	58.45
P*σ_GB_* (%)	25.81	31.12	26.45	17.64	15.49	14.76
*σ_d_* (MPa)	393.55	426.6	623.12	203.31	229.69	249.29
P*σ_d_* (%)	61.87	60.68	68.55	63.22	61.41	62.95
